# Fertility-Related Concerns in Long-Term Survivors of Childhood Cancer: A Canadian Cohort Study

**DOI:** 10.3390/curroncol31120560

**Published:** 2024-11-30

**Authors:** Pauline Holmer, Brianna Henry, Jenny Duong, Oluwaseyi A. Lawal, Miranda M. Fidler-Benaoudia, Kathleen Reynolds, Gisela Michel, Vicky Lehmann, Fiona S. M. Schulte

**Affiliations:** 1 Faculty of Health Sciences and Medicine, University of Lucerne, 6002 Lucerne, Switzerland; pauline.holmer.1@uni-potsdam.de (P.H.); gisela.michel@unilu.ch (G.M.); 2 Department of Oncology, Division of Psychosocial Oncology, University of Calgary, Calgary, AB T2S 3C3, Canada; brianna.henry@ucalgary.ca (B.H.); jenny.duong1@ucalgary.ca (J.D.); oalawal@ucalgary.ca (O.A.L.); 3 Department of Medical Psychology, Amsterdam University Medical Center, University of Amsterdam, 1105 AZ Amsterdam, The Netherlands; v.lehmann@amsterdamumc.nl; 4 Cancer Center Amsterdam, 1081 HV Amsterdam, The Netherlands; 5 Faculty of Human Sciences, Department of Inclusive Education, University of Potsdam, 14476 Potsdam, Germany; 6 Department of Cancer Epidemiology and Prevention Research, Cancer Care Alberta, Alberta Health Services, Calgary, AB T2N 5G2, Canada; miranda.fidler-benaoudia@albertahealthservices.ca; 7 Departments of Oncology and Community Health Sciences, University of Calgary, Calgary, AB T2S 3C3, Canada; 8 Long Term Survivor’s Clinic, Alberta Children’s Hospital, Calgary, AB T3B 6A8, Canada; kareynol@ucalgary.ca; 9 Department of Family Medicine, University of Calgary, Calgary, AB T2N 1N4, Canada

**Keywords:** childhood cancer, survivorship, long-term outcomes, fertility-related concerns, reproductive health

## Abstract

Survivors of childhood cancer face reduced fertility, which can be a significant cause for concern. Our study aimed to assess the prevalence of fertility-related concerns and identify associated factors. Self-report data were collected with the Long-Term Survivor Questionnaire at the Alberta Children’s Hospital’s Long-Term Survivor Clinic (LTSC) between January 2021 and September 2023. Eligible participants were diagnosed before the age of 21 years, ≥2 years off therapy, and ≥13 years old. We analyzed fertility-related concerns cross-sectionally for the whole sample and longitudinally for a subsample of participants that completed three questionnaires. We included *N* = 311 survivors (49.2% female; mean age = 22.7 years, range = 13.9–42.1; mean time since diagnosis = 14.5 years, range = 2.7–38.4), of whom 21.2% reported fertility-related concerns. Those with additional health concerns and those who were sexually active were more likely to report fertility-related concerns, whereas lymphoma survivors were least likely to report concerns. In the subsample who completed three questionnaires (*n* = 80), 30% reported having concerns at least once, whereas 9% expressed persistent concerns. Fertility-related concerns are highly prevalent among young survivors of childhood cancer and warrant attention from healthcare professionals. Services that systematically address fertility-related concerns throughout long-term follow-up are urgently needed. These services should provide a space to discuss concerns, provide education, and initiate fertility consultations if desired.

## 1. Introduction

Although improvements in treatments have significantly increased childhood cancer survival in recent decades [1], survivors remain at risk of multiple late effects, including gonadal dysfunction and infertility [1,2,3,4]. It is estimated that at least 50% of survivors will experience gonadal- or fertility-related late effects in their lifetime [5].

Gonadal- and fertility-related late effects are significant problems, as most childhood cancer diagnoses and treatments occur long before thoughts of potential fertility and family planning. Yet, many survivors of childhood cancer express desires to have biological children in the future [6,7], with some reporting that their cancer experience has increased their desire to have children [8]. As a result, childhood cancer survivors may experience *fertility-related concerns or worries* [9]. Beyond concerns regarding the potential inability to have biological children, fertility-related concerns may involve the risk of miscarriages or complicated pregnancies [10,11], the body’s ability to handle a pregnancy, or the potential impact of fatigue on raising a child [10]. Fertility-related concerns can also center on a potential child’s health, including fears that the child might develop cancer [11,12,13]. Furthermore, they can include concerns about telling a potential partner about fertility and the impact it might have on the relationship [14].

Fertility-related concerns can cause strong emotions like fear, worry, anxiety, sadness, and distress [11,15], and can interfere with sleep and appetite, or distract from everyday activities [11]. Such concerns might also negatively affect romantic relationships, intimacy, and partner communication. Survivors report worrying about disappointing their partner or even feeling guilty about not being able to “provide” a partner with children [7,11,13,14,16]. Some are also concerned, or have already experienced, that telling a partner about potential fertility problems will lead to rejection [7]. Understanding these concerns and addressing them systematically in follow-up care is essential for childhood cancer survivors, as these concerns can have a significant negative impact on their quality of life and interpersonal relationships.

While fertility-related concerns appear distressing to survivors, most of the research on fertility-related concerns in childhood cancer has been qualitative; thus, little is known about the prevalence in large samples and possible associated factors. In addition, the majority of this research has been conducted in the United States, [9] which highlights the need for research in other countries. Therefore, the primary objectives of this study were (1) to assess the prevalence of survivors expressing fertility-related concerns cross-sectionally and (2) to identify sociodemographic, medical, and health-related factors associated with fertility-related concerns in a Canadian cohort of childhood cancer survivors. Finally, we aimed to (3) assess how fertility-related concerns changed over time in survivors who completed multiple questionnaires during the study period.

## 2. Materials and Methods

### 2.1. Study Design and Procedure

Data for this study were collected between January 2021 and September 2023 and the primary outcome of interest was self-reported fertility-related concerns. We used data from a single-center cohort of survivors of childhood cancer in Alberta, Canada. As part of their routine clinic appointment, all survivors were asked to complete a Long-Term Survivor Questionnaire, which assessed their current health status. The questionnaire has received ethics approval from the Health Research Ethics Board of Alberta Cancer Committee (HREBA.CC-19-0498; Edmonton, AB, Canada) and has the primary purpose of facilitating communication between the LTSC team and the survivors/families in order to improve clinical care. We would like to emphasize that the questionnaire was primarily designed for clinical care, not research purposes. The questionnaire is emailed or mailed to all survivors one week prior to their routine clinic appointment. If they do not complete the questionnaire, they receive up to two reminders. Depending on the physician’s risk assessment, survivors come to the clinic twice a year, once a year, or once every two years. The LTSC benefits from having a multidisciplinary team of healthcare professionals, including physicians, nurse practitioners and mental health specialists, who collaborate to provide a comprehensive approach to patient care.

### 2.2. Study Population

Our sample included survivors of childhood cancer enrolled in an LTSC at Alberta Children’s Hospital, Calgary, AB, Canada. Eligible participants were diagnosed before the age of 21 years and had not undergone therapy for at least two years. Furthermore, they were at least 13 years old at time of the study, as this is the age at which survivors are asked to complete self-report versions of the questionnaire. Parent-proxy reports are no longer available at this age, as we have shifted to prioritizing self-reported data from adolescents, who are showing an increasing ability to engage with sensitive topics directly. The survivors consented to have their data used as part of a larger study.

### 2.3. Measurements

Fertility-related concerns were assessed with the following face-valid question: “Do you have any concerns regarding fertility?” (No/Yes). Other items included in the LTSC questionnaire were as follows: “Which best describes your gender identity?” (Female/Male/Prefer not to answer/Prefer to self-describe); “Are you experiencing fear of other [non-cancer] health problems?” (No/Yes); “Are you sexually active?” (No/Yes); “Are you currently having problems with sexual functioning?” (No/Yes); and “Have you ever been pregnant/fathered a child?” (No/Yes). Medical information was obtained from patient information sheets provided by the LTSC and included the type of cancer diagnosis (Leukemias/Solid tumor/Brain tumor/Lymphoma), type of treatment (Surgery/Chemotherapy/Radiation), relapse (No/Yes), age at diagnosis, time since diagnosis and current age.

### 2.4. Statistical Analysis

All statistical analyses were performed using IBM^®^ SPSS^®^ Statistics, Version 29.0. We used descriptive statistics to describe the sample characteristics. Fisher’s exact/χ^2^- and *t*-tests were used to test whether fertility-related concerns (No/Yes) differed by background factors, e.g., age or type of diagnosis, which may influence the main outcome, at a univariate level. Subsequently, background factors that were significantly related to fertility-related concerns were included in a logistic regression to test their relative contribution at a multivariate level. Factors were only included if there were at least 10 cases per predictor (*p*-values of < 0.05 were considered statistically significant).

We then used descriptive statistics to examine how survivors who completed three questionnaires during the study period reported fertility-related concerns over time and grouped them accordingly (never concerned/ever concerned). We conducted Fisher’s exact/χ^2^- and *t*-tests to test whether groups (never concerned/ever concerned) differed by background factors.

## 3. Results

### 3.1. Sample Characteristics

The whole sample consisted of 312 long-term survivors of childhood cancer, of whom *N* = 311 answered the question about fertility-related concerns and were therefore included in this study (Table 1). Participants were nearly evenly split into male and female survivors and had a mean age of 22.7 years (range = 13.9–42.1). The mean age at diagnosis was 8.1 years (range = 0.1–20.8) and the mean time since diagnosis was 14.5 years (range = 2.7–38.4). Most participants were survivors of solid tumors (37.3%) or leukemias (35.7%).

### 3.2. Prevalence of Fertility-Related Concerns and Associated Factors

At completion of the first questionnaire during the study period, 21.2% of the sample (*n* = 66) reported fertility-related concerns. Fear of other health problems (χ^2^(1) = 19.20, *p* < 0.001), problems with sexual functioning (χ^2^(1) = 11.86, *p* = 0.003), and being sexually active (χ^2^(1) = 7.86, *p* = 0.006) were significantly associated with reporting fertility-related concerns. When using a continuous variable, age was not significantly associated with fertility-related concerns. However, when contrasting and comparing adolescents versus adults (as a dichotomous variable), adults were significantly more likely to report fertility-related concerns (χ^2^(1) = 4.20, *p* = 0.047). Furthermore, the type of diagnosis was significant (χ^2^(3) = 8.33, *p* = 0.040); solid tumor survivors were significantly more likely to report concerns than lymphoma survivors. Graphical presentations of the distributions of significant variables are presented in Figure S1. There were no significant differences for age at diagnosis or time since diagnosis (Table 2).

The significant factors listed above (i.e., age at study, type of diagnosis, fear of other health problems, and being sexually active) were included in a logistic regression to test their relative contribution to fertility-related concerns. We also included gender identity, as it was on the borderline of statistical significance. Problems with sexual functioning were not included due to low numbers (*n* = 9). The omnibus test of model coefficients demonstrated overall significance of the logistic regression analysis (χ^2^(7) = 35.79, *p* < 0.001) with an explained variance of 17.4%. Lymphoma survivors were almost four times less likely to report fertility-related concerns compared to solid tumor survivors (OR = 0.26, 95% CI [0.10, 0.68], *p* = 0.006). Individuals who expressed fear of other health problems were three times more likely to report fertility-related concerns than those without such fears (OR = 3.05, 95% CI [1.62, 5.73], *p* < 0.001). In addition, survivors who were sexually active were two times more likely to report fertility-related concerns than those who were not sexually active (OR = 2.00, 95% CI [1.01, 3.98], *p* = 0.048) (Table 3).

### 3.3. Prevalence of Fertility-Related Concerns and Associated Factors over Time

Longitudinal data were available for 25.7% of the sample (*n* = 80), of whom 70.0% never reported fertility-related concerns, 16.0% reported fertility-related concerns once, 5.0% reported such concerns twice, and 9.0% reported such concerns at all time points (Figure S2). Combining these numbers, 30.0% of survivors reported fertility-related concerns at least once. Survivors who have had surgery (χ^2^(1) = 6.92, *p* = 0.010) and survivors who were older at diagnosis (*t*(78) = −2.18, *p* = 0.032) were more likely to report fertility-related concerns (Table S1). Due to the limited sample size of participants who completed multiple questionnaires and the resulting low counts for certain cells when stratifying by background factors, we refrained from conducting multivariable analyses.

## 4. Discussion

Our study demonstrated that fertility-related concerns are highly prevalent among survivors followed by the LTSC in Alberta, Canada. About one-fifth of the sample reported fertility-related concerns at the first measurement point during the study period, but this proportion increased to almost one-third when analyzing responses over time, with 9.0% of survivors expressing persistent fertility-related concerns at all time points. This emphasizes the enduring nature of fertility-related concerns, which may become even more important for survivors who want to have children as they approach their child-bearing years [11], and it highlights an urgent need to address such concerns in clinical care. Nevertheless, survivors may begin to explore the idea of having children long before actually starting a family [17]. This is supported by our results showing that nearly ten percent of adolescents (<18 years old) reported fertility-related concerns. Parents often play a crucial role in fertility-related discussions at the time of diagnosis, serving as advocates and decision-makers, especially when survivors are too young to fully comprehend the long-term implications of their treatment and its impact on their fertility. Their involvement may help establish a foundation of awareness that benefits survivors as they mature and begin to address these concerns independently in survivorship care [18]. Several factors were significantly associated with fertility-related concerns cross-sectionally. Survivors who were afraid of other health problems and survivors who were sexually active were significantly more likely to report fertility-related concerns. Certain survivors may experience increased anxiety, such as worries about late effects and chronic health conditions, or might be more anxious generally, which might explain the association with fertility-related concerns. Accordingly, a previous study reported that survivors who worried about cancer recurrence were also more likely to worry about their fertility [19]. The association between sexual activity and fertility-related concerns may be interpreted in different ways: for instance, it is likely that those in committed relationships are more sexually active and more realistically considering future parenthood with their partners. Consistent with this, survivors in relationships report more fertility-related concerns [7,11,13,14,16]. At the same time, some of the survivors who are in relationships may be actively trying to have a child. The period of trying to conceive as a survivor has been described as very stressful [15] and may also increase fertility-related concerns. Associations between sexual functioning and fertility-related concerns could not be formally tested, but descriptive statistics suggested a relationship. Accordingly, a previous study indicated that more than half of study participants spontaneously discussed fertility-related concerns when asked about sexual function, even though fertility-related topics were not part of the interview guide [16]. Furthermore, lymphoma survivors were less likely to report concerns than survivors of solid tumors. More research is needed to determine how different cancer types are associated with fertility-related concerns.

In our longitudinal analyses, being older at diagnosis was significantly associated with fertility-related concerns. Survivors who were older at diagnosis may have been more involved in fertility discussions at the time of diagnosis and may therefore be more aware of their increased infertility risk. Moreover, survivors who had undergone surgery were more likely to report concerns, but analyses were limited by subgroups with n < 5 and should thus be interpreted with caution. Nevertheless, both variables have previously been shown to have a negative impact on health-related quality of life [20,21], which may as well be related to fertility concerns; this topic requires further research.

Although quantitative studies on this topic are limited, existing studies report prevalence rates of fertility-related concerns among childhood cancer survivors that are comparable to our findings [13,22,23]. Fertility-related concerns may not be inherently negative. Being informed about potential fertility problems or an increased likelihood of infertility can help to inform survivors’ decisions regarding their reproductive goals, family planning, and conversations with (potential) partners. Unfortunately, survivors generally appear to have insufficient knowledge about infertility as a possible side effect of cancer treatment, and show a tendency to underestimate the risks [8,24]. Moreover, survivors are typically unaware of their fertility status (with 77.1% of survivors being unaware of their fertility status [6]), as the majority of survivors do not undergo fertility testing [25]. Typically, survivors only find out their fertility status when they/their partner get pregnant, rather than as a result of a fertility assessment [7]. Alarmingly, reported individual risk perceptions can be highly discordant with the actual fertility risk [24,26,27]. This may lead to fertile survivors worrying unnecessarily and at-risk survivors not worrying “enough”, and therefore possibly not exploring alternative options in time.

Such discrepancies underscore the importance of survivorship care in this domain. However, fertility consultations can also increase reproductive concerns [22]. It is therefore important to offer nuanced and good-quality fertility counseling rather than just ensuring availability [22]. The goal of discussing fertility with providers should be to review the potential late effects of cancer treatment, address concerns, provide education, and, if desired, offer a referral for a fertility assessment, including gonadotropin levels and semen analyses for males and gonadotropin levels, antimullarian hormone testing as well as transvaginal ultrasounds for females. Although the test results may be difficult to interpret (especially for female survivors), if infertility is suspected, survivors should be adequately supported and provided with information exploring alternative paths to parenthood, if desired. However, care should be taken not to offer alternatives to biological parenthood (e.g., adoption) as an “easy” alternative, as such options can come with great obstacles for cancer survivors. Earlier research suggests that survivors may lack information about the lengthy and expensive procedures required for adoption [15].

Fertility (preservation) discussions have been cited as a criterion for high-quality childhood cancer care [28], and counseling is related to increased knowledge about fertility [8]. Unfortunately, previous studies suggest that many survivors perceive fertility discussions as unsatisfactory or do not recall ever being informed about their increased risk of infertility [29]. If they do recall being provided with such information, they describe fertility discussions as challenging because of providers’ lack of knowledge and/or dismissal of their concerns [11]. Some survivors have reported feeling ignored and stated that the absence of discussions further contributed to their concerns [30]. Furthermore, the costs associated with assisted reproduction can be prohibitive for survivors [31]. Unmet needs regarding fertility-related information can lead to a reduced quality of life in survivors [32]. LTSC healthcare providers play a critical role in meeting these needs, as survivors expect them to initiate conversations [33]. However, healthcare providers should be aware of and sensitive to the fact that not all survivors want to have children [7,10].

Beyond talking with providers, survivors may benefit from other forms of support, such as online discussion forums and peer support, which can provide a sense of understanding and belonging [10].

### Strengths and Limitations

As mentioned in the introduction, survivors’ fertility-related concerns can extend beyond fertility itself and include aspects such as partner communication [10,11,12,13,14]. Our question “Do you have any fertility-related concerns?” is rather broad and therefore leaves room for interpretation. Furthermore, we lack information about survivors’ fertility status and their knowledge about it. Due to some smaller subgroups with certain background characteristics, we were unable to include some potentially relevant factors in our analyses. For example, we were unable to analyze survivors who did not identify as female or male, even though they are an important group to consider when talking about fertility-related concerns [34]. In addition, the frequency of clinic visits varied among survivors, with some attending twice a year while others visited once a year or once every two years. As a result, the limited number of participants who completed multiple questionnaires within the study timeframe led to analyses that remain exploratory and require further research. Although our questionnaire included basic sociodemographic factors, it did not assess how broader social conditions might influence survivors’ access to fertility-related information, which is an important area for future research. Overall, a more comprehensive approach to assessing fertility-related concerns (e.g., surveys including open-ended questions and interviews) will help to clarify the exact nature of the reported concerns. In the future, we also want to include providers’ perspectives and consider survivors’ explicit needs to better understand how fertility-related concerns can ideally be addressed in our follow-up clinic.

This is the first study to examine the fertility-related concerns of childhood cancer survivors living in Alberta, Canada. It allowed us to determine the prevalence of fertility-related concerns and to identify associated factors. Importantly, we were able to assess fertility-related concerns over time to better understand the potential persistent nature of concerns. We also consider it a strength that we were able to include so many male survivors in our sample. Furthermore, we included adolescent and adult survivors in our sample. Overall, our study will inform future research and help to further develop our survivorship program.

## 5. Conclusions

Fertility-related concerns are highly prevalent among childhood cancer survivors and warrant attention from healthcare providers. The high prevalence, early onset, and persistence of fertility-related concerns underscore the importance of discussing fertility repeatedly throughout the survivorship journey. The development of programs to systematically address fertility-related concerns in long-term follow-up is urgently needed.

We believe that systematically providing fertility education (e.g., information on the potential impact of cancer treatments on fertility), offering tailored discussions (e.g., personalized guidance on fertility preservation options) and making fertility testing available would not only help survivors manage their concerns but would also empower them to make informed decisions about their reproductive health. Based on our results, we recommend that these programs begin early and be provided regularly. However, we acknowledge that further research is needed to refine and evaluate such interventions, as this was beyond the scope of our current study.

## Figures and Tables

**Table 1:** Sample characteristics (N = 311).

	N (%) ^a^	Mean (SD)	Range
Age at study in years		22.7 (6.0)	13.9–42.1
Age group at study			
Adolescents: 13–17 years	71 (22.8)		
Adults: ≥18 years	237 (76.2)		
Gender identity			
Female	153 (49.2)		
Male	151 (48.6)		
Non-binary	2 (0.6)		
Prefer not to answer	3 (1.0)		
Age at diagnosis in years		8.1 (5.4)	0.1–20.8
Time since diagnosis in years		14.5 (6.2)	2.7–38.4
Diagnosis			
Leukemias	111 (35.7)		
Brain tumors	25 (8.0)		
Lymphoma	59 (19.0)		
Solid tumors	116 (37.3)		
Treatment			
Surgery	200 (64.3)		
Chemotherapy	296 (95.2)		
Radiation	121 (38.9)		
Relapse	34 (10.9)		

^a^ Missing values were ≤1% for all variables and are therefore not reported separately.

**Table 2:** Factors associated with fertility-related concerns (N = 311).

	Fertility-Related Concerns				
	No (*n* = 245, 78.8%)	Yes (*n* = 66, 21.2%)			
	*n* (%)	*n* (%)	Fisher’s Exact Test (*p*)		
Age group			**0.047**		
Minor (0–17.99)	62 (87.3)	9 (12.7)			
Adult (>18)	180 (75.9)	57 (24.1)			
Gender identity ^a^			0.050		
Female	113 (73.9)	40 (26.1)			
Male	126 (83.4)	25 (16.6)			
Diagnosis			**0.040** ^b^		
Solid tumor	82 (70.7)	34 (29.3)			
Leukemias	91 (82.0)	20 (18.0)			
Brain Tumor	20 (80.0)	5 (20.0)			
Lymphoma	52 (88.1)	7 (11.9)			
Surgery			0.081		
Yes	151 (75.5)	49 (24.5)			
No	93 (84.5)	17 (15.5)			
Radiation			0.088		
Yes	89 (73.6)	32 (26.4)			
No	155 (82.0)	34 (18.0)			
Fear of other health problems			**<0.001**		
Yes	41 (59.4)	28 (40.6)			
No	200 (84.0)	38 (16.0)			
Problems with sexual functioning			**0.003**		
Yes	3 (33.3)	6 (66.7)			
No	241 (80.6)	58 (19.4)			
Ever been pregnant or fathered a child			0.464		
Yes	22 (84.6)	4 (15.4)			
No	179 (77.2)	53 (22.8)			
Sexually active			**0.006**		
Yes	112 (72.3)	43 (27.7)			
No	133 (85.3)	23 (14.7)			
	**Fertility-related concerns**				
	**No (*n* = 245)**	**Yes (*n* = 66)**			
	**Mean (SD)**	**Mean (SD)**	***t* (df)**	***p***	**Cohen’s d**
Age at study	22.4 (6.0)	23.9 (5.8)	−1.81 (306)	0.071	0.251
Age at diagnosis	8.0 (6.3)	8.3 (6.1)	−0.35 (93) ^c^	0.724	0.053
Time since diagnosis	13.7 (5.8)	14.7 (7.4)	−1.02 (88) ^c^	0.312	0.162

^a^ Those who did not identify as female or male were counted as missing; ^b^ χ^2^-test; ^c^ equal variances were not assumed because Levene’s test was significant; bold values indicate statistically significant results (*p* < 0.05).

**Table 3:** Logistic regression (χ^2^(7) = 35.79, Nagelkerke R^2^ = 0.174, *p* < 0.001), including significant factors of primary analyses (N = 299) ^a^.

Variables	*OR* [95% CI]	*p*-Value
Age group		
Minor	1	
Adult	1.38 [0.56, 3.40]	0.490
Gender identity		
Male	1	
Female	1.65 [0.91, 3.00]	0.103
Diagnosis		
Solid Tumor	1	
Brain Tumor	0.76 [0.25, 2.33]	0.628
Leukemias	0.56 [0.29, 1.08]	0.083
Lymphoma	0.26 [0.10, 0.68]	**0.006**
Fear of other health problems		
No	1	
Yes	3.05 [1.62, 5.73]	**<0.001**
Sexually active		
No	1	
Yes	2.00 [1.01, 3.98]	**0.048**

^a^ N = 12 survivors were excluded from the logistic regression due to missing values; bold values indicate statistically significant results (*p* < 0.05).

## Data Availability

The data that support the findings of this study are available from the corresponding author upon reasonable request.

## Supplementary Materials

The following supporting information can be downloaded at https://www.mdpi.com/article/10.3390/curroncol31120560/s1, Figure S1: Distribution of fertility-related concerns by significant background factors; Figure S2: Prevalence of fertility-related concerns over time (*N* = 80); Table S1: Factors associated with fertility-related concerns over time (*N* = 80).
