# Supplementary material for: Fertility-Related Concerns in Long-Term Survivors of Childhood Cancer: A Canadian Cohort Study
Source: Curr Oncol. 2024 Nov 30;31(12):7603–12. doi: 10.3390/curroncol31120560 (PMC11674506; doi:10.3390/curroncol31120560)
Supplement: Supplementary file 1 [file curroncol-31-00560-s001.zip › curroncol-3266372-supplementary.pdf]

Overview

Supplementary Figure S1 - Distribution of fertility-related concerns by significant background factors

Supplementary Figure S2 - Prevalence of fertility-related concerns over time (*N*=80)

Supplementary Table S1 - Factors associated with fertility-related concerns over time (*N*=80)

Supplementary Figure S1 - Distribution of fertility-related concerns by significant background factors

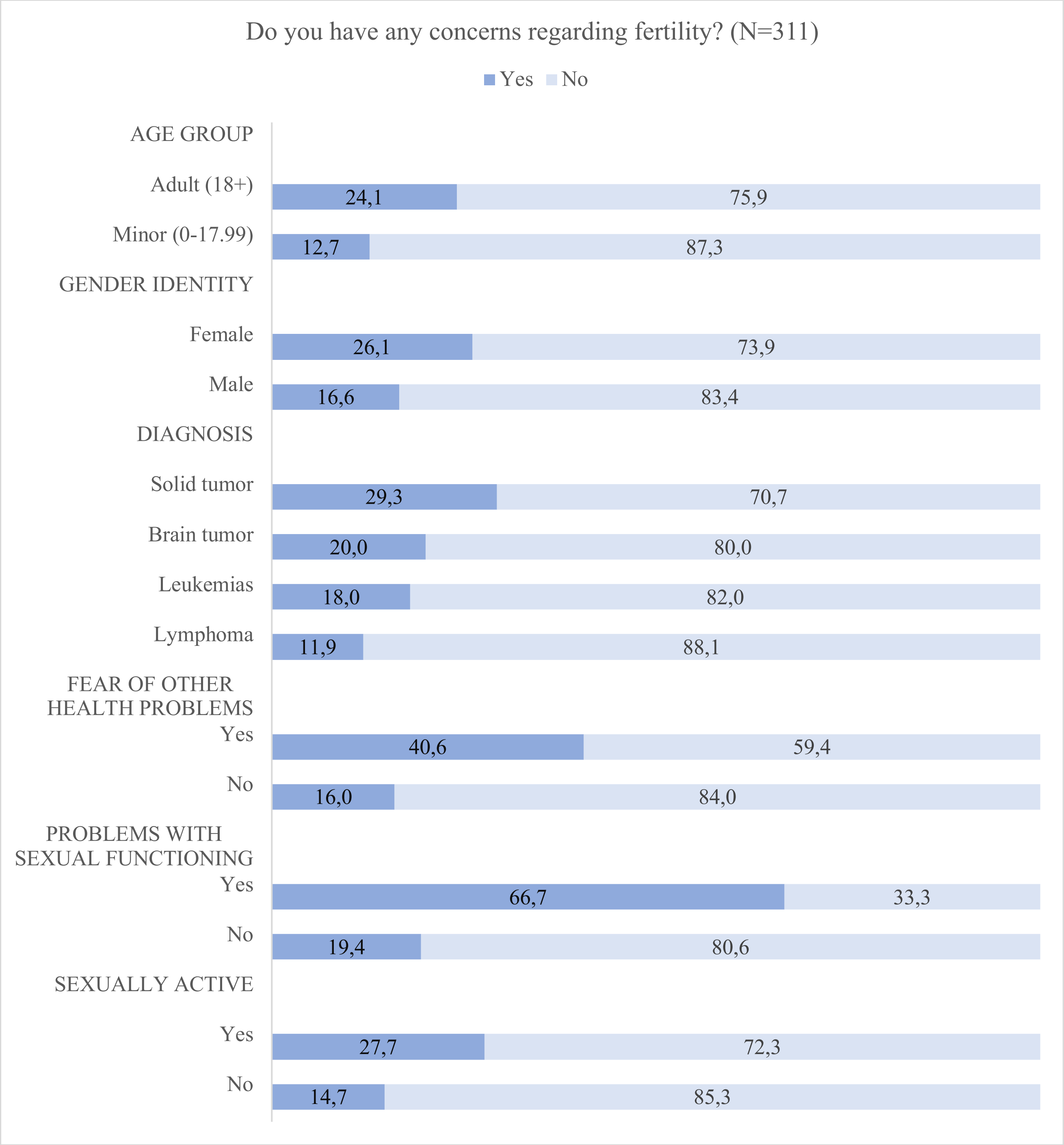

Supplementary Figure S2 - Prevalence of fertility-related concerns over time (*N*=80)

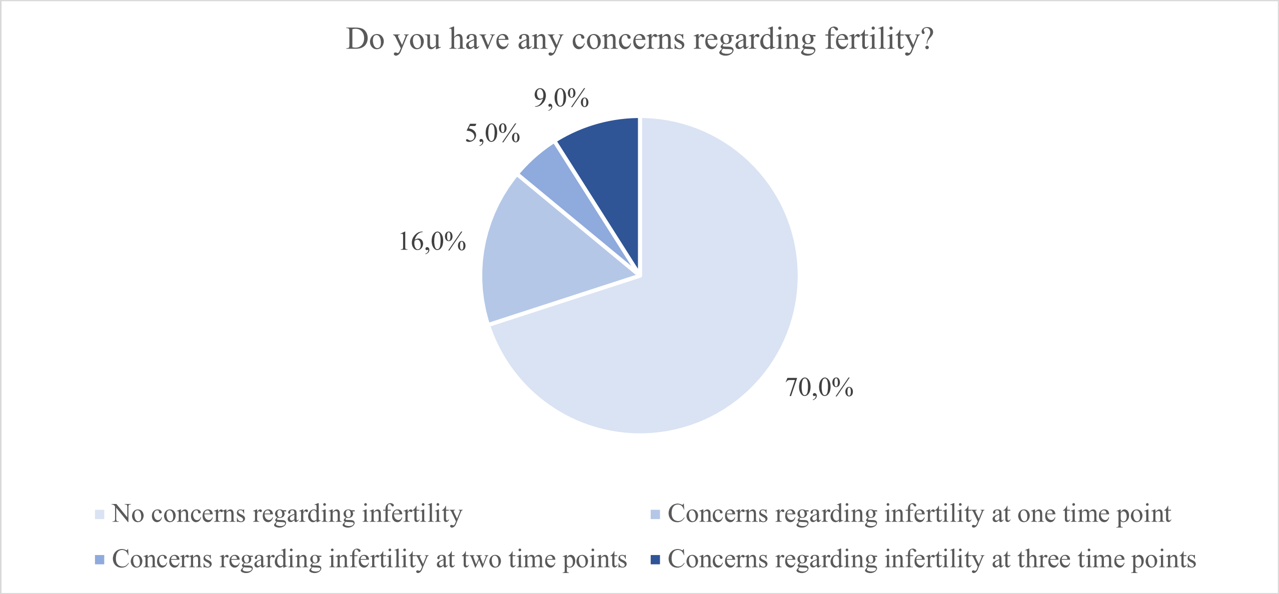

Supplementary Table S1 - Factors associated with fertility-related concerns over time (*N*=80)

	Fertility-related concerns		
	Never (*n*=56)	Ever (*n*=24)	
	n (%)	*n* (%)	Fisher's exact test (*p*)
Gender identity			.465
Female	24 (64.9)	13 (35.1)	
Male	31 (73.8)	11 (26.2)	
Diagnosis			.074[a]
Solid tumor	18 (58.1)	13 (41.9)	
Leukemias	21 (84.0)	4 (16.0)	
Brain Tumor	4 (50.0)	4 (50.0)	
Lymphoma	13 (81.3)	3 (18.8)	
Surgery			**.010**
Yes	32 (60.4)	21 (39.6)	
No	24 (88.9)	3 (11.1)	
Radiation			.219
Yes	21 (61.8)	13 (38.2)	
No	35 (76.1)	11 (23.9)	
Fear of other health problems			.616
Yes	22 (66.7)	11 (33.3)	
No	34 (73.9)	12 (26.1)	
Problems with sexual functioning			.358
Yes	3 (50.0)	4 (50.0)	
No	53 (71.6)	21 (28.4)	
Ever been pregnant or fathered a child			
Yes	7 (70.0)	3 (30.0)	1.0
No	49 (70.0)	21 (30.0)	
Sexually active			.325
Yes	29 (64.4)	16 (35.6)	
No	26 (76.5)	8 (23.5)	

	Fertility-related concerns				
	Never (*n*=56)	Ever (*n*=24)			
	Mean (SD)	Mean (SD)	*t*(df)	*p*	Cohen's d
Age at last questionnaire	24.6 (5.8)	26.6 (5.3)	-1.44 (77)	.153	.353
Age at diagnosis	7.2 (5.5)	10.3 (6.4)	-2.18 (78)	**.032**	.531
Time since diagnosis at last questionnaire	17.3 (4.5)	15.9 (7.6)	1.02 (77)	.409	.249

[a] X^2-test; Note: Variables highlighted in gray contain cell frequencies n<5.; Background factors were calculated based on responses from any time point throughout the study period, bold values indicate statistically significant results ($p < 0.05$)
